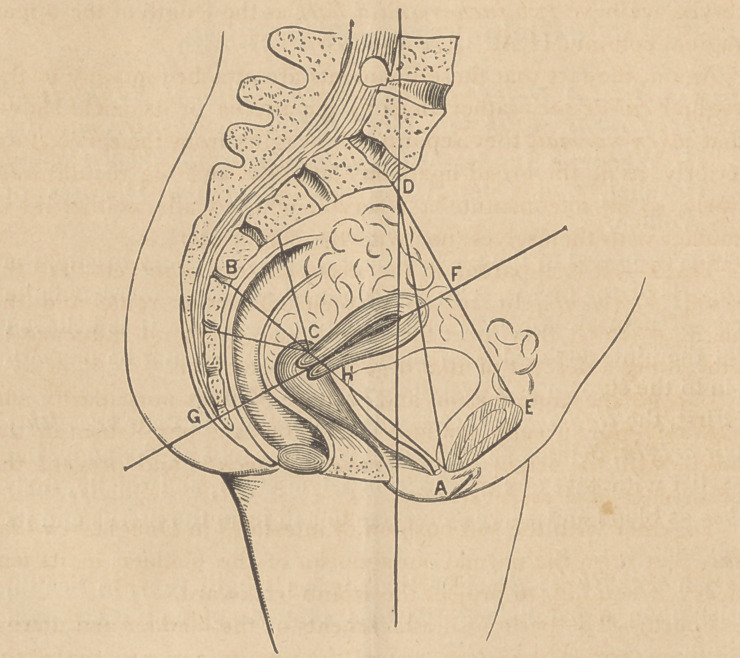# Brief Review of the Natural Supports of the Uterus

**Published:** 1870-01

**Authors:** H. Webster Jones

**Affiliations:** Accoucheur to Cook County Hospital, and Clinical Lecturer upon the Diseases of Women


A MONTHLY RECORD OF

*Medicine, Surgery, and the Collateral Sciences.*

EDITED BY J. ADAMS ALLEN, M.D., LL.D.; AND WALTER HAY, M.D.

---

VOL. XXVII.—JANUARY, 1870.—No. 1.

---

### Original Communications.

ARTICLE I.—*A Brief Review of the Natural Supports of the Uterus, with Certain Inferences therefrom.* By H. WEBSTER JONES, M.D., Accoucheur to Cook County Hospital, and Clinical Lecturer upon the Diseases of Women.

IT is proposed in the present paper, to review, very briefly, the supports of the uterus, with the hope of securing more definite ideas of nature's purposes concerning that organ. It is believed that the hints deduced from such an inquiry may not be unavailable to the student and practitioner.

First, the *ligamenta lata* interest in respect to their *breadth*; their *origin*, oblique as compared with the axis of the body, but parallel with that of the superior pelvic strait, and finally, their *office* as blood and nerve conveyers to and from the uterus and its appendages.

By a *breadth* as great as the uterine length they, in health, insist upon a parallelism between that viscus and their own origin.

Their obliquity, therefore, determines the direction of the uterine axis, while their central origin preserves its body from an infringement, on the one hand, upon the bladder, and on the other, upon the *cul-de-sac*, whose intestinal contents should form one of its strongest safeguards.

The *office* of these ligaments, when retroversion of the uterus has occurred, is interfered with, much as if their vessels and nerves had been subjected to "torsion" by the forceps of the surgeon.

Second — The *cervico-sacral* ligaments are of great importance, as conservators of the uterine balance.

These are notable in respect to their *line of tension*, their *normal length*, and finally, their *attachment*, which is less to the uterus itself than to the *vaginal cul-de-sac*.

Applying the law of the "composition of forces" to these ligaments, they are found to form, together with the upper vaginal wall, a *chord*, whose arc is composed of the sacral curve (below the third bone), the coccyx and perineal muscles, ending in the sub-pubic ligament.*

* The above Diagram is based, geometrically, upon the researches of Professor Hodge, who seems to have devoted much time and pains to the attainment of exact ideas, in regard to the relative distance and place of the pelvic structures and organs.

A line A C B represents the chord mentioned, and A G B is its arc. The former will be found very nearly coincident with the superior vaginal wall, A H, its line of cervical attachment, H C, and the line C B, representing the direction of the composite cervico-sacral force.

The *normal length* of these supports must evidently conform to the distance of the sacrum from the intersecting transverse plane of the pelvis, in which the *broad ligaments* lie, less half the thickness of the *cervix uteri.* The line B C indicates this distance.

In a standard pelvis, Professor Hodge gives the diameter A C B as four inches and eight lines. Professor Meigs (Diseases of Woman, p. 208) says that the distance of the cervix from the sacrum ought to be from *one* to *one and a half inches*, and that the vaginal length (measured upon the line A C) should be *three inches and a half.* Allowing one inch as the thickness of the cervix, we have *two inches and a half* as the length of the upper vaginal column, H A.

Again, the fact that these ligaments are attached mainly to the vaginal *cul-de-sac*, rather than to the uterus or its neck, shows that *when normal*, they separate the vagina from the cervix, posteriorly, as do the broad ligaments laterally, and the rectum inferiorly, so as to constitute a reservoir whose walls neither lie in contact with themselves, nor with the cervix uteri.

Third — It is apparent to the most cursory observer, that the *round ligaments*, by reason of their circuitous route and the angle at which they meet the uterus, can exert no influence in preventing a descent of that organ in its own axis.

But, for the same reason, and because of their muscularity and elasticity, they greatly conduce to the constant parallelism of the uterus with the origin of the *broad ligaments*, and prevent the occurrence of a *retroversion.*

Together with the soft cushion of intestines in Douglas' *cul-de-sac*, they form the normal antagonism of the bladder, in its tendency, when full, to project the womb backward.

Fourth—The peritoneal attachments of the bladder and uterus, by some termed the *utero-vesical ligaments*, serve to make the movements of these organs, to a large extent, mutual, and explain some of the sympathies which exist between them.

Fifth — The *vagina* and *perineal muscles* are important adjuvants to the uterine balance.

The former is such simply and *only* because it forms part of the chord A H C B.

By the elasticity of its upper wall, it preserves a due extension of the *cervico-sacral ligaments* and the cervix, at a proper distance from the arc A G B. The distance F G (or the pelvic *depth*) is stated by Professor Hodge, as four inches and six lines, and he gives the length of the uterus as two inches and a half.

Allowing six lines for the distance between the plane of the superior strait and the *fundus uteri*, we then have, approximately, one inch and a half as the elevation of the *os uteri* above the arc mentioned.

Given the pelvic diameters as stated, and the uterine and vaginal dimensions, as indicated by the above well-known authorities, and it is impossible for the vagina to meet the uterus otherwise than at an *acute angle;* it cannot, then, form a "columnar pedestal," upon which the latter may rest.

The *levator ani* muscle, in transit from its anterior origins, embraces the vagina obliquely, just behind its proper sphincter.

By a few muscular fibres sent off to the upper wall of that canal, it assists in maintaining proper tension; and, by similar union with the lower wall, it conduces to the apposition of the two.

The perineal muscles cannot act *directly* upon the womb, unless that organ has *lapsed* a full inch and a half, or lies *retroverted* upon the rectum.

They form a *corps de reserve*, useful indeed, in all violent depressions of the abdominal and pelvic contents from external force.

The pelvic fascia and interstitial cellular tissue contribute to the general strength and safety of the supports enumerated.

The following reflections are incident upon this view of the subject:

1st. The uterus is intended to observe certain relationships to other organs; in fact, to possess a *normal position*, having variations which are limited in extent.

2nd. The uterus, like other bodies whose main support is below their centre of gravity, is liable to divergence from its normal position.

3rd. Anatomically, it is least protected from an *anteversion* (or flexion), for the uterine axis is obliquely forward, as regards the force of gravity, and it sustains, upon its posterior and upper wall, some of the weight of the intestines. Moreover, there are no *fundo-sacral* ligaments; and the bladder, generally empty, or nearly so, is the sole antagonist of the round ligaments.

Among one hundred and fourteen women examined, by M. Panas (*vide* "L'Union Medicale"), one-third were subjects of these errors in place; and they were mainly young, unmarried or non-parturient, and therefore, less liable to accident.

Inferentially, this diversion should be attended by less physical annoyance than its opposite state. Torsion of the *broad ligaments* can here only reach over an arc of 35°, and is not necessarily obstructive.

4th. In her defences against *retroversion*, nature has spared no pains. The round ligaments above and in front, and the cervico-sacral below and behind, not to speak of the obliquity of the broad ligaments and the intestinal compress in Douglas' *cul-de-sac*, all fortify the uterus most evidently, in this direction. The cervix kept within an inch and a half of the sacrum, and the round ligaments preserving a fundo-pubic distance of two inches and a half, no retroversion *can* occur. (Meigs, Woman and Her Diseases, p. 208.)

Inferentially, this displacement is far more serious in its results than any other. Torsion of the broad ligaments may here extend over an arc of 160°, obstructing the venous channels, giving rise to congestions, ovarian and uterine, and by impeded or distorted reflex action, originating hypertrophies, hyperæsthesias, and disurbances of the menstrual function.

Such is the success of fashion, as arrayed against nature, that "seventy-five per centum of uterine disorders and displacements consist in retroversion of the womb," (*loc. cit.*)

5th. The uterus can descend in its own axis, (*i. e.*, "*lapse*") but *one inch and a half.*

It can be moved forward, its obliquity being preserved ("*prolapse*") not more than two inches and a half.*

* The writer considers that any divergence of the upper uterine axis backward, so far as, or beyond a parallelism with the vertical axis of the body, derivatively speaking, a "*retroversion.*"

6th. Inferentially, the *os uteri* was not intended to impinge upon the pelvic floor.

Still less should it rest upon foreign bodies of greater firmness and resistance.

7th. The vagina is intended as a *reservoir.*

Retroversion and prolapse rob it of its receptive and retentive powers, and diminish the probability of conception.

Anteversion has less of this tendency.

8th. The fact that the *cervix uteri* "falls easily" into the mouth of a speculum, is corroborative of a tendency to retroversion, the normal angle of incidence of uterus upon vagina being about 70° — an *acute* angle.

9th. No pessary should fix the womb *immovably*, or elevate the *os uteri* more than one inch and a half (to two inches?) above the perineal structures, or force the *cervix* backward more than two inches and a half from the pubis. The point C (*vide* diagram) may be thus held at a distance of three and a half inches.

Any abdominal compress, however constructed or applied, forces the intestines in the direction where there is least resistance; will inevitably and in time overcome the elasticity and tone of the uterine supports, muscular or ligamentous, causing *lapse* and *prolapse*, and when a degree of *retroversion* exists, will certainly increase its extent, and hasten the evils which attend thereupon.

Finally, artificial supports for the uterus should always harmonize with Nature's provision for the safety of tissues, the propagation of the species, and the comfort, bodily and mental, of the patient wearer.

## Figures and Tables

**Figure f1:**